# CCL19-armed recombinant influenza virus inhibited colorectal cancer growth by remodeling tumor microenvironment

**DOI:** 10.1016/j.isci.2025.114127

**Published:** 2025-11-19

**Authors:** Xia Ou, Yunxin Xia, Zhongyue Fang, Kai Yang, Guangtao Yang, Junying Wang, Yiyong Duan, Xiahui Yang, Bing Yang, Ze Liu, Jihong Zhang

**Affiliations:** 1 Basic Medical School, Kunming University of Science and Technology, Kunming 67800, P.R. China; 2 Library, Kunming Medical University, Kunming 678000, P.R. China; 3 Lijiang People’s Hospital, Lijiang 674100, P.R. China; 4 School of Basic Medical Science, Tianjin Medical University, Tianjin 300400, P.R. China; 5 Department of Public Health, International School, Krirk University, Bangkok, Thailand; 6 Bio-X Center for Interdisciplinary Innovation, Yunnan University, Kunming 678000, P.R. China; 7 School of Life Sciences, Tianjin University, Tianjin 300401, P.R. China

**Keywords:** Immune system, Immunology specialty, Cancer

## Abstract

The “cold” tumor microenvironment (TME) impaired colorectal cancer (CRC) immunotherapy efficacy, while oncolytic viruses (OVs) could “heat” TME and stimulate anti-tumor immunity. Here, it rescued *r*PR8-CCL19, a recombinant oncolytic influenza virus expressing CCL19, using reverse genetics technology. Gene sequencing and transmission electron microscopy confirmed its genetic stability during serial passaging. Hemagglutination assay, ELISA, transwell, real-time cell analysis (RTCA), and apoptosis detection demonstrated that it selectively infected CRC cells, expressed CCL19 with the function of chemotaxis and activation on immune cells, and exerted killing of significant CRC cells. In syngeneic CRC mouse models, it effectively inhibited tumor growth and metastasis, prolonging survival. By enhancing immune cell infiltration, it remodeled the TME, thereby inducing systemic anti-tumor immunity and even immune memory, without causing severe pathological damage. This study confirmed *r*PR8-CCL19 as a promising CRC immunotherapy for further exploration.

## Highlights

• rPR8-CCL19 shows selective infection and potent cytotoxicity in CRC cells
• Stable rPR8-CCL19 expresses CCL19, driving DC and T cell recruitment and activation
• rPR8-CCL19 inhibits CRC growth/metastasis and induces robust systemic immune memory
• rPR8-CCL19 remodels the tumor microenvironment via immune cells and cytokines

## Graphical abstract

**Figure:**
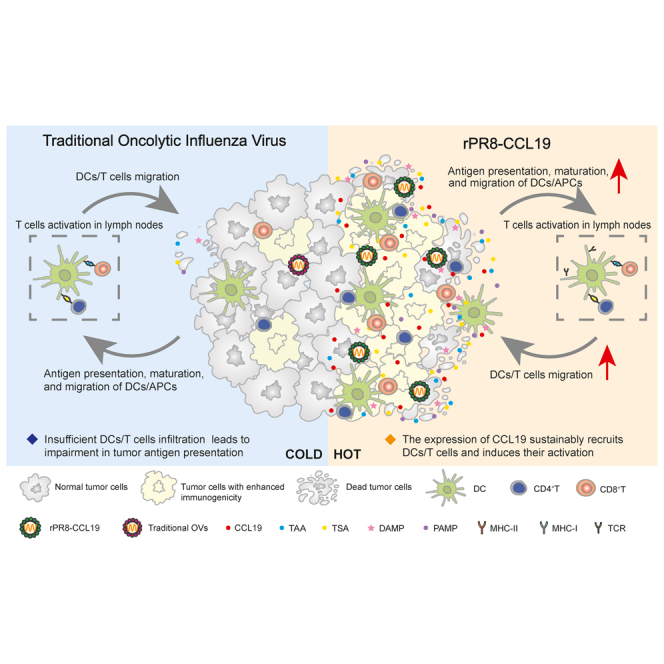

## Introduction

Colorectal cancer (CRC) was a major public health challenge, which was the third most impact cancer and the second leading cause of cancer-related mortality worldwide. Traditional treatments are surgery, chemotherapy, and radiotherapy; however, their side effects, e.g., recurrence, nausea, diarrhea, nervous system damage, blood system dysfunction, and radiation enteritis, might negatively impact clinical outcomes.^1^^,^^2^ In 2015, a significant breakthrough was achieved with the introduction of immune checkpoint inhibitors (ICIs) to treat CRC.^3^ However, it is important to note that PD-1 antibody monotherapy was effective in only 10% of patients with high microsatellite instability (MSI-H), while the remaining 90% of patients with microsatellite stability (MSS) and low microsatellite instability (MSI-L) could not benefit from that immunotherapy.^4^^,^^5^ The primary reason for this was that MSS and MSI-L CRC were categorized as “cold” tumors, which had lower tumor-infiltrating T lymphocytes, especially cytotoxic T lymphocytes (CTLs), in the tumor microenvironment (TME). Ultimately, it led to patients having almost no response to the treatment with ICI. Consequently, strategies aimed at recruiting and activating tumor-infiltrating T lymphocytes would remold the immunosuppressive TME, thereby enhancing the effectiveness of immunotherapy for patients with MSS and MSI-L CRC is necessary.

Oncolytic virus (OV) therapy has shown great promise in the field of tumor immunotherapy. Notably, in 2015, the FDA approved talimogene laherparepvec, a recombinant HSV-1 expressing granulocyte-macrophage colony-stimulating factor, for the treatment of melanoma.^6^ Subsequently, in 2021, DELYTACT, an oncolytic HSV-1 virus developed by Daiichi Sankyo Co., Ltd., received approval in Japan for the treatment of glioblastoma and other brain cancers.^7^ OVs were a kind of genetically modified viruses capable of selectively infecting and lysing tumor cells while sparing normal cells. OVs exert their anti-tumor effects primarily through infection and lysing tumor cells directly and serving as carriers for the expression of foreign genes, which could enhance oncolytic and immunostimulant activities.^8^^,^^9^

The influenza virus, as an oncolytic vector, has garnered increasing attention due to its several advantages.^10^^,^^11^ First, the influenza virus was a single negative-stranded RNA virus devoid of reverse transcriptase and DNA integration activity. Furthermore, the high expression of N-acetylsialic acid, a receptor binding to influenza virus hemagglutinin (HA), was widespread on the surfaces of various tumor cells, especially lung cancer, CRC, and prostate cancer. Additionally, the influenza virus has contributed to robust immunogenicity and immunomodulatory capabilities. Consequently, the influenza virus could be a promising vector for OV therapy.

On the other side, CCL19, also known as macrophage pro-inflammatory protein 3-β (MIP3-β), primarily binds to its receptor CCR7 and serves as a core driver for the homing of dendtric cells (DCs), T cells to central immune organs, peripheral lymph nodes, and inflammatory sites. Recent studies have unveiled the critical role of CCL19 in maintaining T cell homeostasis, facilitating the endocytosis of DCs, and promoting NK cell proliferation.^12^

It should be pointed out that high expression of CCL19 in solid tumors not only plays an important role in recruiting T cells to TME through lymphocyte homing but also enhances the activity of T cells, leading to remodeling of the TME and significant improvement in prognosis.^13^^,^^14^ Furthermore, CCL19 as a mono-therapeutic agent was limited due to its short half-life, high permeability, and potent toxic effects.^13^ Therefore, it became imperative to employ vectors for delivering CCL19 into the TME, allowing for its stable expression within tumor tissues and the provision of long-term anti-tumor effect.

In this study, we employed the influenza A virus strain, A/Puerto Rico/8/34 (PR8), as the genetic framework of an oncolytic influenza virus. We strategically inserted the CCL19 orthologous genes in humans and mice, at the 3′ end of the PB1 gene of PR8, and successfully rescued a new oncolytic influenza virus carrying CCL19 that inhibited the CRC growth through remodeling TME, which was named as *rPR8-CCL19*, by reverse genetic manipulation. That *r*PR8-CCL19 exhibited dual surprising capabilities: efficient replication within CRC cells, which led to tumor cell lysis; and these CCL19 expressions could recruit and activate lymphocytes for remodeling TME. Our research encompassed a comprehensive evaluation of the biological characteristics of this new oncolytic influenza virus, confirming its anti-tumor efficacy and safety both *in vitro* and *in vivo*. Additionally, it initiated an exploration of its underlying anti-tumor mechanisms. This study not only laid the foundation for the development of innovative OV but also provided a new perspective for the treatment of CRC.

## Results

### Generation and identification of rPR8-CCL19

**Figure 1:**
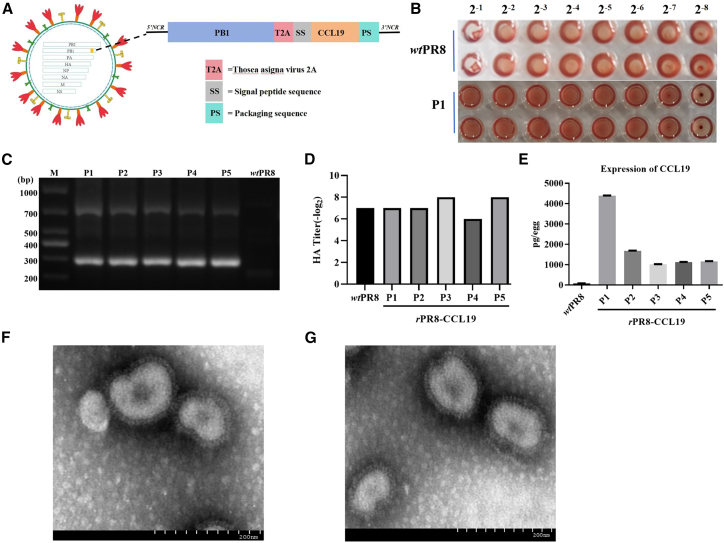
Generation and characterization of recombinant PR8-CCL19 (*r*PR8-CCL19) (A) Schematic design of *r*PR8-CCL19 via insertion of the CCL19 gene into the 3′ terminal region of the PB1 gene of the PR8 influenza virus strain. (B) The H&A titer of P1 *r*PR8-CCL19 (1:128) detected by hemagglutination assay. P1, passage 1 of *r*PR8-CCL19; *wt*PR8, wild-type PR8. (C) Real-time PCR amplification of CCL19 gene (target fragment size:∼700 and ∼300 bp) was to identify genetic stability of *r*PR8-CCL19. M, 1,000 bp DNA marker; P1–P5, passage 1–5 of *r*PR8-CCL19; *wt*PR8, wild-type PR8. (D) Quantification of HA titers of P1–P5 *r*PR8-CCL19. Data were analyzed with descriptive statistical method and represented as mean ± SEM. *n* represents technical replicates, *n* = 3. (E) The CCL19 expression and secretion in chicken embryos infected with P1–P5 *r*PR8-CCL19 detected through ELISA. Data were analyzed with descriptive statistical method and represented as mean ± SEM. *n* represents technical replicates, *n* = 3. (F) Transmission electron microscopy image showed the morphology of *r*PR8-CCL19. Scale bars, 200 nm. (G) Transmission electron microscopy image showed the morphology of *wt*PR8. Scale bars, 200 nm.

The construction diagram of the new oncolytic influenza virus encoding CCL19, which was named as *r*PR8-CCL19, is shown in Figure 1A. Its hemagglutination (HA) titer could reach 1:128 from the first to fifth passages (from P1 to P5) in the chick embryo (Figures 1B and 1D). The right size of the target gene was confirmed by real-time PCR (Figure 1C), which was then confirmed by gene sequencing. ELISA results showed that the expression of CCL19 was the highest at its first passage, almost reaching 4,200 pg/egg, while it remained stable between 1,000 and 1,500 pg/egg from P2 to P5 passages (Figure 1E).

Additonally, *r*PR8-CCL19 observed under transmission electron microscopy revealed mostly spherical virus particles with distinct spike protein structures on surface (Figure 1F) and their particle size ranged predominantly between 80 and 120 nm. Its morphology and size were almost consistent with the wild-type PR8 (*wt* PR8) (Figure 1G).

Overall, these findings demonstrated that *r*PR8-CCL19 has been successfully rescued; it maintained high replication efficiency, genetic stability, effect expression of CCL19, and morphological integrity during its continuous passage.

### rPR8-CCL19 infected CRC cells and inhibited their growth

**Figure 2:**
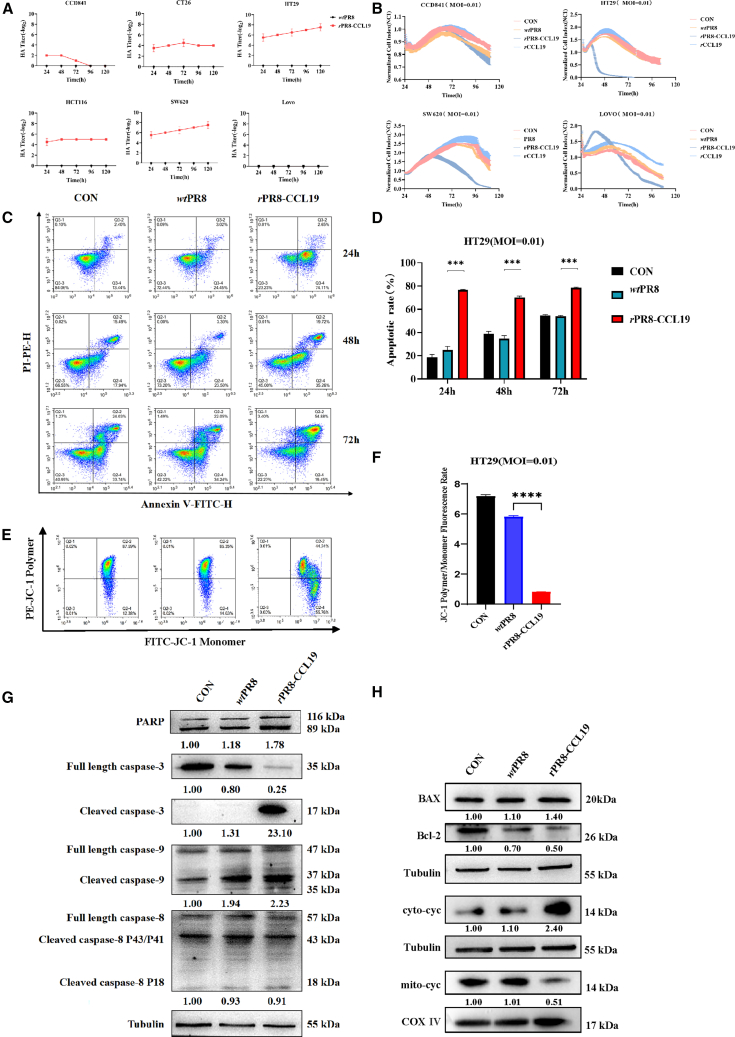
The replication and tumor cell-killing activity of *r*PR8-CCL19 *in vitro* (A) The proliferation dynamics of *r*PR8-CCL19 in various CRC cell lines (CT26, HT29, HCT116, SW620, and Lovo) and the normal colonic mucosal epithelial cell line CCD841, as determined by the HA titter measurement. Data were analyzed with descriptive statistical method and represented as mean ± SEM. *n* represents technical replicates, *n* = 3. (B) Cell killing study performed through xCELLigence RTCA representing individual cell impedance measurements of cells in real time in response to the treatment with *r*PR8-CCL19, *wt*PR8, and *r*CCL19, respectively. The control group (CON) with non-treatment was also applied. (C) The flow cytometric analysis of apoptosis in HT29 cells infected with *r*PR8-CCL19 and *wt*PR8 at 24, 48, and 72 h post-infection, respectively. The CON with non-treatment was also applied. (D) Quantification of HT29 cells apoptotic ratio. *r*PR8-CCL19 induced more than 78% cell apoptosis at 24 h post-infection. Data were analyzed with unpaired two-sided Student’s *t* test and represented as mean ± SEM. *n* represents technical replicates, *n* = 3. ∗*p* < 0.05, ∗∗*p* < 0.01, ∗∗∗*p* < 0.001, ∗∗∗∗*p* < 0.0001. (E) Detection of changes in mitochondrial membrane potential (MMP) in HT29 cells at 24 h post-infection with *r*PR8-CCL19 by flow cytometry, compared with *wt*PR8 and CON. (F) Quantification of MMP in HT29 cell. MMP decreased significantly after *r*PR8-CCL19 treatment. Data were analyzed with unpaired two-sided Student’s *t* test and represented as mean ± SEM. *n* represents technical replicates, *n* = 3. ∗*p* < 0.05, ∗∗*p* < 0.01, ∗∗∗*p* < 0.001, ∗∗∗∗*p* < 0.0001. (G) The expressions of PARP, caspase-3, caspase-9, caspase-8, and their corresponding cleavers in HT29 cells infected with *r*PR8-CCL19 or *wt*PR8 were detected by western blotting, respectively. (H) The expressions of BAX, Bcl-2, cyto-cyc (cytochrome in cytoplasm), and mito-cyc (cytochrome in mitochondria) in HT29 cells infected with *r*PR8-CCL19 or *wt*PR8 were detected by western blotting, respectively.

The high-efficiency infection and replication ability of the oncolytic influenza virus in tumor cells were prerequisites for effectively targeting and killing tumor cells. The CRC cells (HT29, SW620, HCT116, Lovo, and CT26) and the normal colorectal epithelial cell (CCD841) were employed to evaluate the oncotropism and infectivity effect of *r*PR8-CCL19. These CRC cells were infected with *r*PR8-CCL19 (MOI = 0.01) for varying durations (24, 48, 72, 96, and 120 h, respectively). The results showed that the infection and replication efficiencies of *r*PR8-CCL19 were notably higher in HT29, SW620, HCT116, and CT26 cells, in which the HA titer could reach above 1:32 after 24 h (Figure 2A).

The xCELLigence RTCA assay was performed to detect the viability of cells after OV treatment. The results revealed that *r*PR8-CCL19 presented high inhibitory efficacy on HT29, SW620, and Lovo cell growth, with half or more cell viability inhibited after infection with an MOI = 0.01 virus for 96 h. However, it found less inhibitory effect toward CCD841, while *wt*PR8 had no significant effect on cell infectivity under the same conditions (Figure 2B). Additionally, results from the Cell Titer-Glo Assay further supported that *r*PR8-CCL19 could inhibit the CRC cell growth, particularly in HT29, CT26, and HCT116 cells (Figure S1A).

On the other hand, HT29 cells were infected by the virus at an MOI = 0.01, and apoptotic cell phenotypes were detected by flow cytometry. Results demonstrated that *r*PR8-CCL19 induced significant apoptosis with about 70% after 24 h post-infection but *wt*PR8 could not (Figures 2C and 2D). A similar trend was observed in CT26 cells (Figures S1B and S1C).

As for the molecular pathway exploration, the western blot experiment revealed significant up-regulation of cleaved PARP, caspase-3, and caspase-9 in HT29 cells infected with *r*PR8-CCL19 (MOI = 0.01) for 24 h compared to the *wt*PR8 group and PBS control group (Figure 2G). Additionally, a similar treatment effect was also observed in CT26 cells (Figure S1D). These findings indicated that *r*PR8-CCL19 could induce the apoptosis of CRC cells, whose oncolytic capability remained consistent across different cell lines, maintaining its therapeutic efficacy against different types of CRC. The expressions of BAX and cytochrome *c* in the cytoplasm were significantly up-regulated, while Bcl-2 in the cytoplasm and cytochrome *c* in mitochondria were down-regulated (Figure 2H). Flow cytometry results showed that the mitochondrial membrane potential (MMP) was significantly decreased after HT29 cells were infected with *r*PR8-CCL19 (MOI = 0.01) for 24 h (Figures 2E and 2F). These findings indicated that *r*PR8-CCL19 could induce the apoptosis of CRC cells through the mitochondrial apoptosis pathway.

### rPR8-CCL19 could mediate the over-expression of CCL19 in tumor cells and chemotax/activate immune cells *in vitro*

**Figure 3:**
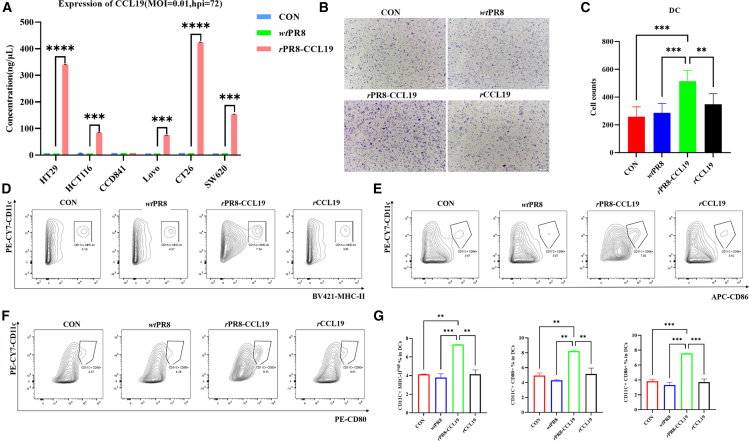
The abilities of *r*PR8-CCL19 expressing CCL19, chemotacticing immune cells, and activating them *in vitro* (A) The expression and secretion of CCL19 were detected by ELISA in various colorectal cancer cells infected with *r*PR8-CCL19 at 72 h post-infection. Data were analyzed with unpaired two-sided Student’s *t* test and represented as mean ± SEM. *n* represents technical replicates, *n* = 3. ∗*p* < 0.05, ∗∗*p* < 0.01, ∗∗∗*p* < 0.001, ∗∗∗∗*p* < 0.0001. (B) Chemotactic effect of CCL19 secreted by rPR8-CCL19-infected colorectal cancer cells on immature dendtric cells (*i*DCs) was detected by transwell assay. (C) Quantification of the number of *i*DCs migrating to the lower chamber in the transwell assay. Data were analyzed with one-way ANOVA and represented as mean ± SEM. *n* represents technical replicates, *n* = 3. ∗*p* < 0.05, ∗∗*p* < 0.01, ∗∗∗*p* < 0.001, ∗∗∗∗*p* < 0.0001. (D) The CD11c^+^MHC-II^+^ DCs were analyzed using flow cytometry. (E) The CD11c^+^CD86^+^ DCs were analyzed using flow cytometry. (F) The CD11c^+^CD80^+^ DCs were analyzed using flow cytometry. (G) Quantitative analysis of CD11c^+^MHC^+^II DCs, CD11c^+^CD86^+^ DCs, and CD11c^+^CD80^+^ DCs, respectively. Data were analyzed with one-way ANOVA and represented as mean ± SEM. *n* represents technical replicates, *n* = 3. ∗*p* < 0.05, ∗∗*p* < 0.01, ∗∗∗*p* < 0.001, ∗∗∗∗*p* < 0.0001.

To investigate the expression of CCL19 in CRC cells, the cells, including HT29, SW620, HCT116, Lovo, CT26, and CCD841, were infected with *r*PR8-CCL19 (MOI = 0.01) for 72 h, and the supernatants were collected for detecting CCL19 content through ELISA testing. The result showed that the expression of CCL19 was high in HT29, HCT116, LOVO, CT26, and SW620 cells. Particularly in HT29 and CT26 cells, the secreted CCL19 content reached 340 and 423 pg/mL, respectively (Figure 3A).

As a chemokine, CCL19 plays the chemotactic function on various immune cells to induce them to migrate. To investigate this biological function of CCL19 expressed by tumor cells infected with *r*PR8-CCL19, a transwell assay was performed. The results revealed a significantly higher number of immature DCs (*i*DCs) in the *r*PR8-CCL19 group compared to the *wt*PR8 group and the control group (Figures 3B and 3C). A similar effect on macrophages was detected (Figures S2A and S2B).

To explore the ability of *r*PR8-CCL19 to activate immune cells, CT26 cells were infected with *r*PR8-CCL19 (MOI = 0.01) for 48 h, and the supernatant was collected to stimulate *i*DCs for 24 h. The phenotypes were detected by flow cytometry to evaluate the proliferation and differentiation of *i*DCs. It found a significant increase in the levels of CD80, CD86, and MHC-II in the *r*PR8-CCL19 treatment group compared to other groups (Figures 3D–3G). These findings suggested that *r*PR8-CCL19 could effectively activate *i*DCs and induce them to differentiate into mature DCs (*m*DCs).

These findings suggested that *r*PR8-CCL19 could not only mediate the over-repression of CCL19 in CRC cells, which exhibited strong chemotactic effects on immune cells, but also activate immune cells *in vitro*.

### rPR8-CCL19 exerted anti-tumor and anti-metastatic effects on CRC effectively and safely *in vivo*

**Figure 4:**
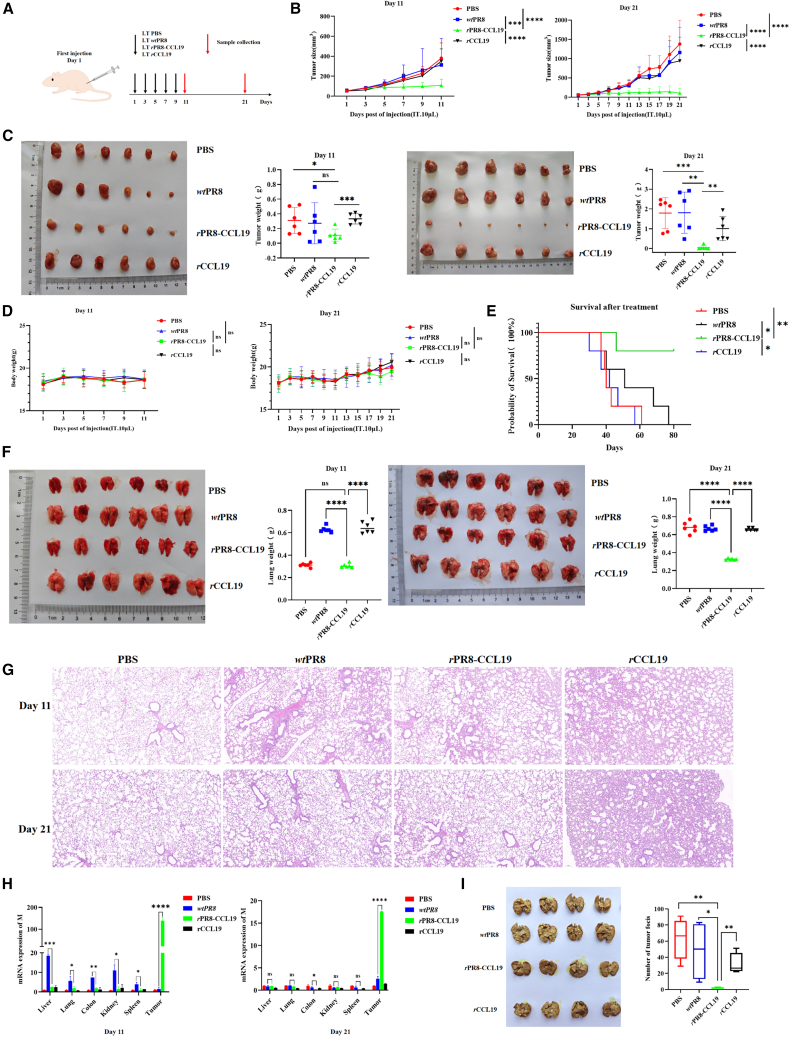
Evaluation of *r*PR8-CCL19 anti-tumor activity and safety in unilateral CT26-bearing mouse model (A) Schedule of the animal experiment using the CT26 tumor model. (B) Tumor volume growth curves of subcutaneous transplanted tumors of CT26 in BABL/C mice treated with PBS (10 μL/mouse), *wt*PR8 (10 μL 1 × 10^5^ PFU/mouse), *r*PR8-CCL19(10 μL 1 × 10^5^ PFU/mouse) and *r*CCL19(0.2μg/mouse), respectively, after 11 and 21 days post-treatment. Data were analyzed with two-way ANOVA and represented as mean ± SEM. *n* represents the number of animals, *n* = 6. ∗*p* < 0.05, ∗∗*p* < 0.01, ∗∗∗*p* < 0.001, ∗∗∗∗*p* < 0.0001. (C) The macroscopic images of the subcutaneous transplanted tumors and corresponding tumor weights. Data were analyzed with one-way ANOVA and represented as mean ± SEM. n represents the number of animals, *n* = 6. ∗*p* < 0.05, ∗∗*p* < 0.01, ∗∗∗*p* < 0.001, ∗∗∗∗*p* < 0.0001. (D) The body weight changes curves of tumor-bearing BABL/C mice after 11 and 21 days post-treatment. Data were analyzed with two-way ANOVA and represented as mean ± SEM. n represents the number of animals, *n* = 6. ∗*p* < 0.05, ∗∗*p* < 0.01, ∗∗∗*p* < 0.001, ∗∗∗∗*p* < 0.0001. (E) The Kaplan-Meier survival curves of tumor-bearing mice in four experiment groups over an 80-day observation. Data were analyzed with the Kaplan-Meier method. n represents the number of animals, *n* = 6. ∗*p* < 0.05, ∗∗*p* < 0.01, ∗∗∗*p* < 0.001, ∗∗∗∗*p* < 0.0001. (F) The macroscopic images of lungs from tumor-bearing mice and corresponding lung wet weight. Data were analyzed with one-way ANOVA and represented as mean ± SEM. n represents the number of animals, *n* = 6. ∗*p* < 0.05, ∗∗*p* < 0.01, ∗∗∗*p* < 0.001, ∗∗∗∗*p* < 0.0001. (G) The histological sections of mouse lung tissues at 11th and 21st day post-treatment were stained by H&E. *r*PR8-CCL19 could not cause pathological injury. Scale bars, 100 μm. (H) The viral bio-distribution at 11th and 21st day post-treatment in tumor-bearing mice by real-time PCR for detection of influenza virus M gene expression *in vivo*. Data were analyzed with unpaired two-sided Student’s *t* test and represented as mean ± SEM. *n* represents the number of animals, *n* = 6. ∗*p* < 0.05, ∗∗*p* < 0.01, ∗∗∗*p* < 0.001, ∗∗∗∗*p* < 0.0001. (I) The inhibitory effect of *r*PR8-CCL19 on lung metastatic CT26 tumors and quantification of the number of tumor foci per mouse. Data were analyzed with one-way ANOVA and represented as mean ± SEM. *n* represents the number of animals, *n* = 4. ∗*p* < 0.05, ∗∗*p* < 0.01, ∗∗∗*p* < 0.001, ∗∗∗∗*p* < 0.0001).

Based on the observed tumor cell-killing efficacy described earlier, it further explored the oncolytic effects of *r*PR8-CCL19 *in vivo* using a CT26 mouse colon cancer model. The administration schedule is shown in Figure 4A, and the mice were sacrificed and the samples were harvested on 11th and 21st day post-treatment, respectively. Significant reduction in tumor size and weight was detected in the *r*PR8-CCL19 group compared with other groups (Figures 4B and 4C). Although the body weight of mice in all groups did not decrease significantly (Figure 4D), *r*PR8-CCL19 improved the mouse survival rate significantly (Figure 4E).

The lung wet weight showed that *r*PR8-CCL19 did not cause significant pulmonary edema but *wt*PR8 and *r*CCL19 did (Figure 4F). Hematoxylin and eosin (H&E) staining also confirmed that *r*PR8-CCL19 had no significant pathological lung injury, while *wt*PR8 and *r*CCL19 induced moderate lung edema and inflammation (Figure 4G). Herein, no significant injury was detected in other organs, including the colon, spleen, liver, and kidney, in all groups both on the 11th and 21st days (Figures S3A and S3B).

To investigate the anti-metastasis effect of rPR8-CCL19, CT26 cells infected with an MOI = 0.01 *r*PR8-CCL19 were injected through the tail vein of mice to establish the tumor lung metastasis model. The results showed a significantly lower number of tumor foci in lung tissue in the *r*PR8-CCL19 group compared to the other two groups (Figure 4I). These results indicate that *r*PR8-CCL19 can inhibit not only the growth of CRC but also the metastasis of CRC cells effectively and safely.

To investigate the bio-distribution of *r*PR8-CCL19 virus loading after treatment, M gene of the influenza vector was detected by real-time PCR. The results showed that although its loading in tumor decreased on the 21st day compared to it on the 11th day, *r*PR8-CCL19 was mainly concentrated in tumor and was almost undetectable in other tissues; while *wt*PR8 wildly distributed in various tissues on the 11th day, it was largely undetectable in all tissues on the 21st day (Figure 4H). The results indicated that *r*PR8-CCL19 might exhibit superior targeting ability toward tumor cells and was less prone to premature clearance by anti-viral immune system, potentially allowing for a prolonged anti-tumor effect.

### rPR8-CCL19 remodeled TME

**Figure 5:**
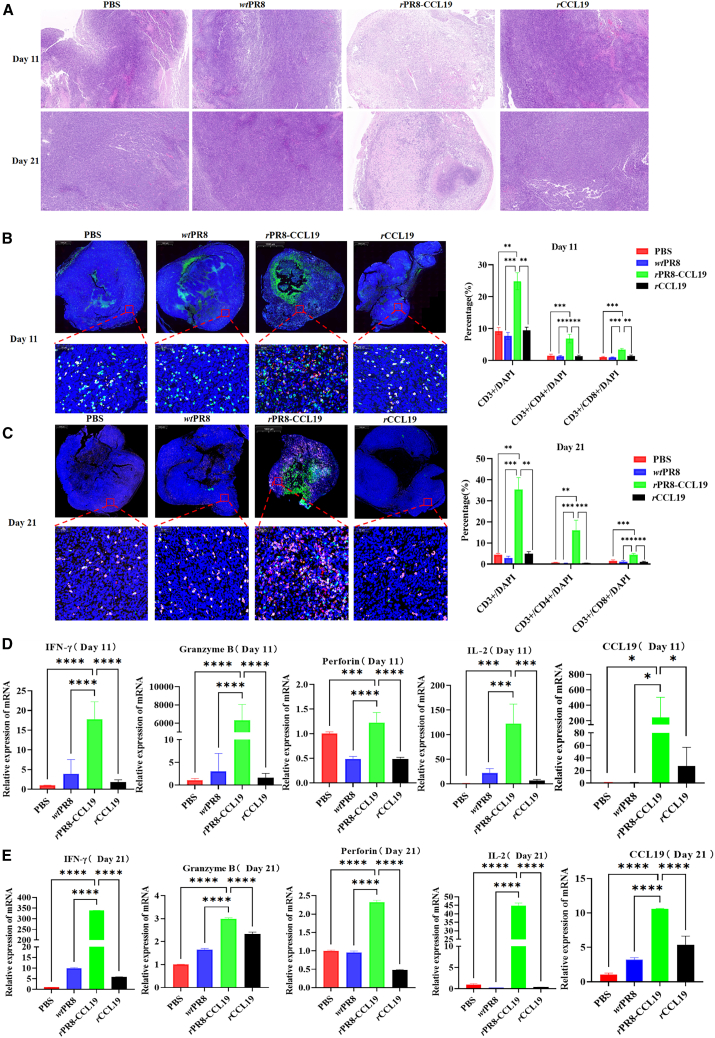
The TME was remodeled by *r*PR8-CCL19 virus after oncolytic treatment *in vivo* (A) Histological sections of tumor tissues were stained by H&E. Scale bars, 100 μm. (B) Histological sections of tumor tissues were stained with anti-CD8 (pink), anti-CD4 (red), and anti-CD3 (green) antibodies for the fluorescence microscopy (scale bars, 1000 μm/50 μm), along with the corresponding quantification of the ratio of total T cells, CD4^+^ T cells, CD8^+^ T cells infiltrating in the TME at the 11th day post-treatment. Data were analyzed with one-way ANOVA and represented as mean ± SEM. *n* represents the number of animals, *n* = 6. ∗*p* < 0.05, ∗∗*p* < 0.01, ∗∗∗*p* < 0.001, ∗∗∗∗*p* < 0.0001. (C) Histological sections of tumor tissues were stained with anti-CD8 (pink), anti-CD4 (red), and anti-CD3 (green) antibodies for the fluorescence microscopy (scale bars, 1000 μm/50 μm), along with corresponding quantification of the ratio of total T cells, CD4^+^ T cells, CD8^+^ T cells infiltrating in TME at the 21st day post-treatment. Data were analyzed with one-way ANOVA and represented as mean ± SEM. *n* represents the number of animals, *n* = 6. ∗*p* < 0.05, ∗∗*p* < 0.01, ∗∗∗*p* < 0.001, ∗∗∗∗*p* < 0.0001. (D) The mRNA expression of Granzyme B, perforin, IL-2, IFN-γ, and CCL19 in TME was detected by real-time PCR at the 11th day post-treatment. Data were analyzed with one-way ANOVA and represented as mean ± SEM. *n* represents the number of animals, *n* = 6. ∗*p* < 0.05, ∗∗*p* < 0.01, ∗∗∗*p* < 0.001, ∗∗∗∗*p* < 0.0001. (E) The mRNA expression of Granzyme B, perforin, IL-2, IFN-γ, and CCL19 in TME was detected by real-time PCR at the 21st day post-treatment. Data were analyzed with one-way ANOVA and represented as mean ± SEM. *n* represents the number of animals, *n* = 6. ∗*p* < 0.05, ∗∗*p* < 0.01, ∗∗∗*p* < 0.001, ∗∗∗∗*p* < 0.0001.

Recruitment and activation of tumor-infiltrating T lymphocytes were key factors in anti-tumor immunity. To investigate this function of *r*PR8-CCL19, H&E staining and *m*IF were performed. Pathological findings revealed that there were fewer tumor cells, which presented diffuse distribution, mild fibrous hyperplasia, and significant necrosis occurred in the center of the tumor tissues in the *r*PR8-CCL19 group. Importantly, there was a strong infiltration of inflammatory cells in the *r*PR8-CCL19 group (Figure 5A). The *m*IF assay results showed that the percentage of CD3^+^T cells, CD3^+^CD4^+^T cells, and CD3^+^CD8^+^T cells infiltrating into tumors in the *r*PR8-CCL19 group was significantly higher than the other two groups both on the 11th and 21st days. Furthermore, the proportions of these cells significantly increased in the *r*PR8-CCL19 group from 23.6% to 36.3%, from 6.3% to 16.9%, and from 3.3% to 5.1%, respectively (Figures 5B and 5C).

To verify the tumor-infiltrating T lymphocytes activated by *r*PR8-CCL19, real-time PCR was employed to detect the expression of granzyme B, perforin, interferon (IFN)-γ, interleukin (IL)-2, and CCL19 in tumor tissues. The results showed that the expressions of those pro-immune effector molecules in the *r*PR8-CCL19 group were significantly higher than other groups, respectively, both on the 11th and 21st days (Figures 5D and 5E). The same trends were also observed by the immunohistochemical method (Figures S4A and S4D).

These results indicated that *r*PR8-CCL19, by infecting CRC and expressing CCL19, not only significantly recruited T cells but also activated them, enabling T cells to express anti-tumor molecules that might enhance tumor cell death significantly.

### rPR8-CCL19 induced systemic anti-tumor immunity and anti-tumor immune memory

**Figure 6:**
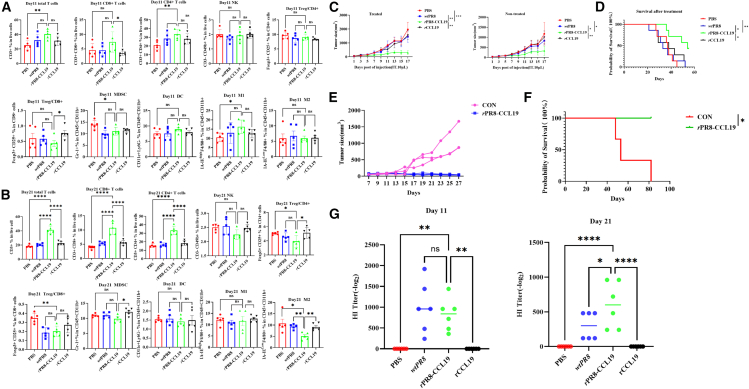
Evaluation of activation of systemic anti-tumor immunity and immune memory *in vivo* (A) The flow cytometry was employed to evaluate the differentiation and development of immune cells in unilateral CT26-bearing mouse models at the 11th day post-oncolytic treatment. Data were analyzed with one-way ANOVA and represented as mean ± SEM. *n* represents the number of animals, *n* = 6. ∗*p* < 0.05, ∗∗*p* < 0.01, ∗∗∗*p* < 0.001, ∗∗∗∗*p* < 0.0001. (B) The flow cytometry was employed to evaluate the differentiation and development of immune cells in unilateral CT26-bearing mouse models at the 21st day post-oncolytic treatment. Data were analyzed with one-way ANOVA and represented as mean ± SEM. *n* represents the number of animals, *n* = 6. ∗*p* < 0.05, ∗∗*p* < 0.01, ∗∗∗*p* < 0.001, ∗∗∗∗*p* < 0.0001. (C) The growth curves of the injected and non-injected tumors (distant tumors). The injected tumors were treated with PBS (10 μL/mouse), *wt*PR8 (10 μL 1 × 10^5^ PFU/mouse), *r*PR8-CCL19(10 μL 1 × 10^5^ PFU/mouse), and *r*CCL19(0.2μg/mouse). The distant tumors were untreated. Data were analyzed with two-way ANOVA and represented as mean ± SEM. *n* represents the number of animals, *n* = 6. ∗*p* < 0.05, ∗∗*p* < 0.01, ∗∗∗*p* < 0.001, ∗∗∗∗*p* < 0.0001. (D) The Kaplan-Meier survival curves of bilateral tumor-bearing mice models in each experimental group. Data were analyzed with the Kaplan-Meier method. *n* represents the number of animals, *n* = 6. ∗*p* < 0.05, ∗∗*p* < 0.01, ∗∗∗*p* < 0.001, ∗∗∗∗*p* < 0.0001. (E) Tumors growth curves in tumor rechallenge models. The mice treated with *r*PR8-CCL19 prevented tumor growth upon re-tumorigenesis due to the induction of robust immune memory. Data were analyzed with descriptive statistical method. *n* represents the number of animals, *n* = 3. (F) The Kaplan-Meier survival curves of tumor re-challenge mice models in the *r*PR8-CCL19 treatment group and CON group. Data were analyzed with the Kaplan-Meier method. *n* represents the number of animals, *n* = 3. ∗*p* < 0.05, ∗∗*p* < 0.01, ∗∗∗*p* < 0.001, ∗∗∗∗*p* < 0.0001. (G) The serum hemagglutination inhibition (HI) titers in each group. *r*PR8-CCL19 and *wt*PR8 groups induced anti-influenza virus immunity at 11th and 21st days after oncolytic therapy in unilateral tumor-bearing mouse models. Data were analyzed with one-way ANOVA and represented as mean ± SEM. *n* represents thne number of animals, *n* = 6. ∗*p* < 0.05, ∗∗*p* < 0.01, ∗∗∗*p* < 0.001, ∗∗∗∗*p* < 0.0001.

Systemic anti-tumor immunity was closely related to tumor regression, metastasis, recurrence and patient prognosis. To evaluate whether *r*PR8-CCL19 could activate systemic anti-tumor immunity, mice spleen cells were prepared for flow cytometry to detect the cell phenotypes and confirm the differentiation and development of immune cells after treatment. It found that the numbers of CD3^+^T, CD3^+^CD4^+^T, and CD3^+^CD8^+^T cells in the *r*PR8-CCL19 group significantly increased, while the number of M2 macrophages significantly decreased on the 21st day (Figures 6A and 6B). It indicated that *r*PR8-CCL19 might activate systemic anti-tumor immunity by inducing Th/CTL cell proliferation and inhibiting M2 macrophage cell proliferation. Furthermore, the results from bilateral mouse CT26 model experiment showed that *r*PR8-CCL19 significantly inhibited tumor growth after different treatment for tumors (Figure 6C) and improved mouse survival significantly (Figure 6D). To investigate the *r*PR8-CCL19 whether induce anti-tumor immune memory, re-challenge study was carried out. The tumor growth was significantly inhibited in *r*PR8-CCL19 group compared with control group (Figure 6E). Additionally, the survival time of mice was significantly prolonged (Figure 6F). These results suggested that *r*PR8-CCL19 could not only recruit and activate immune cells to remodel TME but also induce systemic anti-tumor immunity and immune memory.

It needs to confirm anti-influenza virus immune response was generated *in vivo* after oncolytic therapy and evaluate the impact of this response on the therapeutic efficacy. Experiment mouse serum was collected for hemagglutination inhibition (HI) assays. The results showed that the mean HI titers of anti-influenza virus antibodies in the *wt*PR8 group and *r*PR8-CCL19 group reached 1,000 and 820 on 11th day, respectively, and decreased to 300 and 600 on the 21st day, respectively (Figure 6G). These results indicated that the antiviral immunity induced by *r*PR8-CCL19 was not as strong as that induced by *wt*PR8; however, its duration was longer than that of *wt*PR8. From another perspective, this suggested that *r*PR8-CCL19 exerts an oncolytic effect for a longer period *in vivo*, thereby achieving a better oncolytic outcome.

## Discussion

Oncolytic virus could be a promising immunotherapy method for clinical application due to its advantages of better tumor targeting, various killing pathways to overcome tumor cell specificity.^15^^,^^16^ In this study, we used influenza virus PR8 as a vector to rescue a new oncolytic influenza virus carrying the CCL19 gene, named *r*PR8-CCL19. It could selectively replicate and mediate the expression of CCL19 in various CRC cell lines, with genetic stability. It could not only inhibit CRC growth and metastasis but also recruit and activate T cells to remodel TME. Furthermore, it could trigger systemic anti-tumor immunity and tumor-specific immune memory. Importantly, this therapeutic approach did not induce any significant pathological damage.

In recent years, immune checkpoint blockade (ICB) therapy has assumed a pivotal role as a useful treatment.^3^^,^^15^^,^^17^^,^^18^ However, CRC was a highly heterogeneous disease, and only 10% CRCs present the genetic sub-type characterized by dMMR or MSI-H, which exhibit a high tumor mutation burden, strong tumor immunogenicity, and abundant immune cell infiltration. Patients with dMMR or MSI-H sub-types could derive significant benefits from ICB therapy. However, the remaining 90% CRC patients bearing MSS or MSI-L tumors, which exhibited cold TME, limited tumor immunogenicity, and poor immune cell infiltration, rendering them largely insensitive to ICB, cannot benefit from this treatment.^4^^,^^19^^,^^20^^,^^21^^,^^22^ This disparity in treatment outcomes was primarily attributed to the distinct TME shaped by the genetic mutations. Mounting evidence supported that the sub-type, density, and even the spatial distribution of T cells within TME could serve as predictive markers for clinical outcomes.^23^^,^^24^^,^^25^ Notably, the presence of CTLs within the TME was strongly associated with a more favorable prognosis.^25^ So, enhancing the efficacy of immunotherapy hinges upon elevating CRC immunogenicity and augmenting the infiltration of tumor-specific CTLs to remodel TME.

As known, TME exerted a profound influence on tumor progression and responsiveness to immunotherapy. Many evidence has underscored the pivotal role of chemokines in TME regulation, further influencing tumor initiation, invasion, and metastasis.^26^^,^^27^^,^^28^ The CCL19, a member of the CC chemokine family, emerged as a central player to orchestrate the recruitment of immune cells, such as T cells, NK cells, and DCs, into TME through binding with the CCR7 receptor, subsequently induced the maturation and activation of immune cells. Activated immune cells released a cascade of immune-promoting factors, including IFN-γ, CXCL9/10, IL-12, and GM-CSF, into TME.^12^^,^^29^^,^^30^^,^^31^^,^^32^^,^^33^ Thus intratumoral over-expression of CCL19 emerged as an effective strategy to remodel TME, furthermore, inhibiting tumor growth and metastasis.

However, in this study, there is no significant inhibition activity of CRC cells with the treatment of 1,000 ng/mL *r*CCL19 protein alone *in vitro*. Moreover, when CT26-bearing mice were treated with 0.2 μg *r*CCL19 protein per mouse by intramural injection, it not only failed to induce TIL infiltration and delay tumor growth significantly but also caused serious lung congestion and edema. This might be attributed to the short half-life, high permeability, and strong cytotoxic effect of the CCL19 protein.^34^ Therefore, how to deliver CCL19 into the tumor to make it play a better function of tumor inhibition and reduce its toxic side effects could be a problem that needs to be addressed.

The oncolytic virus had a great function in remodeling TME. They might directly lyse tumor cells and release TAAs, PAMPs, and DAMPs, then recruit endogenous CD3^+^CD4^+^, CD3^+^CD8^+^ effector T cells to infiltrate into TME, and cause the non-immunogenic “cold” tumor to change to the immunogenic “hot” tumor.^35^^,^^36^ Influenza virus, despite its notorious impact on human health, was one of the first viruses to be investigated for cancer therapy—especially in its attenuated type. Through natural breeding or genetic modification, influenza viruses can be developed into attenuated strains with reduced pathogenicity to humans. They can be mass-produced within chicken embryos, utilizing the established influenza vaccine production system. Furthermore, specific antiviral replication drugs, such as oseltamivir, enable the artificial control of virus replication in the body, substantially enhancing the safety of the influenza virus as an oncolytic virus. Adding the advantages described above, the oncolytic influenza virus has garnered significant attention in the field of tumor immunotherapy. Its infection of tumor cells was a critical step in anti-tumor therapy. Studies have revealed that CRC cells can express and secrete trypsin or trypsin-like serine proteases, and they exhibited an over-expression of sialic acid on their cell surface, which was 10–100 times higher than that of normal cells.^37^^,^^38^^,^^39^ This natural affinity of the oncolytic influenza virus for CRC cells suggests significant therapeutic potential. However, it is worth noting that wild-type influenza viruses alone possess limited ability to lyse tumor cells.

In our study, we confirmed that after infecting both normal colorectal mucosal epithelial cells (CCD841) and various CRC cells (including HT29, HCT116, SW620, and CT26) with wtPR8 at an MOI of 0.01 for 72 h, the hemagglutination (HA) titer in the culture supernatant was nearly undetectable. Furthermore, the inhibitory effect of wtPR8 on the viability of these cells was significantly weaker than that of rPR8-CCL19. Therefore, to improve both the oncolytic efficacy and safety profile of the influenza virus, we performed genetic modifications to optimize its anti-tumor potency while reducing the required infectious dose.

The oncolytic influenza virus has shown efficacy not only in immunotherapy for CRC but also exhibited advantages in liver cancer immunotherapy. Yang’s PhD team rescued a chimeric oncolytic influenza virus (rFlu-huCTLA4) carrying a human CTLA4 antibody in the background of the A/PR/8/34 (PR8) virus and found that rFlu-huCTLA4 could selectively destroy hepatocellular carcinoma cells *in vitro* and *in vivo*, potentially providing a promising clinical strategy for targeted immunotherapy of HCC.^40^ In other studies, reverse genetics techniques were exploited to load NA fragments of PR8 with GV1001 peptides derived from human telomerase reverse transcriptase. Their research showed that oncolytic influenza virus significantly inhibited liver tumor growth in mice *in vivo*. Furthermore, it induced an anti-tumor immune response including increased numbers of CD4^+^ and CD8^+^ T lymphocytes which in turn improved survival. These researchers concluded that oncolytic influenza viruses represented a promising immunotherapeutic approach for patients with HCC.^41^

In this study, we utilized the PR8 strain as a viral vector to deliver the chemokine CCL19. The recombinant virus demonstrated efficient replication and transgene expression in chicken embryos. Although an abnormal increase in CCL19 expression was observed in the P1 viral stock, this might be attributed to the transient expression from the recombinant bidirectional plasmid pHW2000-PB1-CCL19 presented in the P0 virus supernatant. Through subsequent serial passages, rPR8-CCL19 exhibited significant genetic stability with no loss of the target gene. The virus showed robust infectivity and replication efficiency in multiple (CRC cell lines—including CT26, HT29, HCT116, and SW620—while displaying limited replicative capacity in normal colorectal mucosal epithelial cells (CCD841).

rPR8-CCL19 induced a higher level of apoptosis across various CRC cell lines compared to the wild-type virus (wtPR8). *In vivo*, rPR8-CCL19 significantly inhibited tumor growth, prolonged survival, and induced tumor-specific immune memory. Analysis by multicolor immunofluorescence and immunohistochemistry revealed that rPR8-CCL19 remodeled the tumor TME, characterized by the robust recruitment and activation of both CD4^+^ and CD8^+^ T cells. This would enhance tumor-specific immune response, especially the biological function of the DC-T cell axis.^42^

On the other hand, results from HI testing showed that both *r*PR8-CCL19 and *wt*PR8 induced specific anti-influenza virus PR8 immune responses *in vivo* after oncolytic therapy. However, the antiviral immunity induced by rPR8-CCL19, though of a longer duration, was weaker than that induced by wtPR8. When combined with viral bio-distribution data, these results suggested that the attenuated systemic immune response might be attributed to the enhanced tumor tropism of rPR8-CCL19, leading to its primary accumulation and prolonged persistence within tumor tissues. Furthermore, tumor growth curves indicated that the antiviral immunity against rPR8-CCL19 had only a limited inhibitory impact on its overall oncolytic efficacy. The underlying mechanisms for this phenomenon need further investigation.

Last, these results from viral bio-distribution, histopathological analysis via H&E staining, the body weight, and survival analysis of mice indicated that *r*PR8-CCL19 exhibited selective replication and safety, without causing significant pathological damage to vital tissues and organs. Thus, *r*PR8-CCL19 might become a safe oncolytic virus therapeutic agent.

This study presented an approach to CRC immunotherapy by engineering an oncolytic influenza virus (*r*PR8-CCL19) to deliver the chemokine CCL19. It was demonstrated that *r*PR8-CCL19 effectively inhibited tumor growth and metastasis, remodeled the TME, and activated anti-tumor immunity in pre-clinical models. The concept was timely and innovative, particularly considering the limitations of current ICB strategies in CRC.

### Limitations of the study

In the future, more science experiments should be taken. It could include a second complementary model, e.g., genetic, chemical, for anti-tumor and anti-metastasis activity, rather than an artificial subcutaneous model. The exclusive use of murine CT26 cells limits generalizability to human CRC. Testing *r*PR8-CCL19 in patient-derived organoids or humanized mouse models would enhance clinical relevance. Optimal viral dosage and administration routes for clinical translation need to be set. A dose-escalation study or comparison of intratumoral vs. systemic delivery would need future trials. The analysis of virus-induced apoptosis, via RTCA/apoptosis assays, could analyze mechanistic validation, e.g., caspase inhibition or necroptosis markers like MLKL phosphorylation.

## Resource availability

### Lead contact

Further information and requests for resources and reagents should be sent directly to the primary contact person, Jihong Zhang (2306371156@qq.com).

### Materials availability

No new unique reagents were generated in this study, and all data in this study are commercially available.

### Data and code availability

• All data reported in this paper will be shared by the lead contact upon request.
• This paper did not report original code.
• Any additional information required to reanalyze the data reported will be shared by the lead contact upon request.

## Acknowledgments

The authors would like to thank all the members who made contributions to this research. This work was supported by the 10.13039/501100001809National Natural Science Foundation of China Project (grant number 82560547), the General project of 10.13039/501100008871Yunnan Province Science and Technology Department (grant nos. 202301AT070478 and 202401AT070369), the 10.13039/501100007301Kunming University of Science and Technology and The First People's Hospital of Yunnan Province Joint Special Project on Medical Research (grant no. KUST-KH2022008Y), and the 10.13039/501100007301Kunming University of Science and Technology and Lijiang People's Hospital Joint Special Project on Medical Research (grant no. KUST-LJ2022004Y).

## Author contributions

Study concept and design, X.O. and Z.L.; acquisition of data, X.O., Y.X., K.Y., Z.F., G.Y., Y.D., and X.Y.; analysis and interpretation of data, X.O., Y.X., J.W., and K.Y.; drafting of the manuscript, X.O., Y.X., B.Y., and Z.L.; critical revision of the manuscript for important intellectual content, X.O., B.Y., and Z.L.; and administrative, technical, or material support and study supervision, X.O. and J.Z.

## Declaration of interests

The authors declare no competing interests.

## STAR★Methods

### Key resources table

**Table:** 

REAGENT or RESOURCE	SOURCE	IDENTIFIER
**Antibodies**		

FITC anti-mouse CD45	Biolegend	103108
PE anti-mouse CD3	Biolegend	100206
APC anti-mouse CD8a	Biolegend	100712
PerCP anti-mouse CD4	Biolegend	100538
PE anti-mouse CD25	Biolegend	102008
APC anti-mouse F4/80	Biolegend	123110
PE anti-mouse CD11b	Biolegend	101208
APC/Cyanine7 anti-mouse Ly-6G	Biolegend	127623
PerCP/Cyanine5.5 anti-mouse Ly-6C	Biolegend	128011
FITC anti-mouse CD4	BioXCell	BE0003-1
BV421 anti-mouse MHC-II	BD Horizon™	562564
APC anti-mouse CD86	Biolegend	105012
PE anti-mouse CD80	BD Pharmingen™	553769
PE anti-mouse CD11c	Biolegend	117318
Flow Cytometry Viability Stain	Biolegend	564997
FITC anti-mouse CD49b	Biolegend	103503
BV421 anti-mouse I-A/I-E	Biolegend	107631
APC anti-mouse Foxp3	Thermo Fisher	77-5775-40
Pro-caspase-3	Abclonal	a19654
Cleaved Caspase-3	CST	9661T
Caspase9/pro-caspase-9	CST	9502T
polyclonal goat anti-Mouse IgG-HRP	Servicebio	GB23301
polyclonal goat anti-Rabbit IgG-HRP	Servicebio	GB23303
rabbit anti-PARP	CST	9532
mouse anti α-Tubulin	Abclonal	AC012
rabbit anti CD8	Servicebio	GB15068
rabbit anti CD4	Servicebio	GB15064
mouse anti CD3	Servicebio	GB12014
mouse anti-Granzyme B	Servicebio	GB12092
rabbit anti- Perforin	ABclonal	A0093
mouse anti-IFN-γ	Thermo Fisher	MM700
CY5 labeled goat anti rabbit IgG	Servicebio	GB25303
CY3 labeled goat anti rabbit IgG	Servicebio	GB21303
Alexa Fluor 488 labeled goat anti mouse IgG	Servicebio	GB25301

**Chemicals, peptides, and recombinant proteins**		

Recombinant Murine IL-4	PeproTech	214-14-100
Recombinant Murine GM-CSF	PeproTech	315-03-250
Hieff® qPCR SYBR Green Master Mix	YEASEN	11201ES03
RDE	Saynher	892021
Eosin Stain	Servicebio	G1001-500 ML
Hematoxylin Stain	Servicebio	G1004-500 ML
0.5% Ammonia Water	Servicebio	G1040-500 ML
DAB Substrate Kit	Abcam	ab64238
Paraffin	Leica	39601095
EDTA	Biosharp	BS107-100g
Tris-base	Solarbio	77-86-1
PrimeSTAR® Max DNA Polymerase	Takara	R045A
Phosphotungstic Acid Negative Stain Solution	Solarbio	G1870

**Critical commercial assays**		

Hifair® AdvanceFast 1^st^ Strand cDNA Synthesis Kit	YEASEN	11149ES60
Hifair® Ⅱ 1^st^ Strand cDNA Synthesis SuperMix for qPCR	YEASEN	11123ES60
Polyfect Transfection Reagent	Qiagen	301105
TIANamp Virus RNA kit	Tiangen	DP315-R
EndoFree Midi Plasmid Kit	Tiangen	DP118
CellTiter-Glo® Luminescent Cell Viability Assay Kit	Promega	G7571
RNAprep Pure Tissue Kit	Tiangen	DP431
Annexin V-FITC/PI Apoptosis Detection Kit	YEASEN	40302ES20
4×Fix/Perm buffer kit	eBiocience	88-8824-00
Mouse CD8^+^ T cell Isolation Kit	BEAVER	70902–50
JC1	Beyotime Biotechnology	C2005
Human CCL19 ELISA Kit	Beijing 4A Biotech	HUEB0059

**Experimental models: Cell lines**		

HT29	ATCC	HTB-38
SW620	ATCC	CCL-227
HCT116	ATCC	CCL-247EMT
Lovo	ATCC	CCL-229
CT26	ATCC	CRL-2638
CCD841	ATCC	CRL-1790

**Experimental models: Organisms/strains**		

BALB/c mice	Vital River Laboratory Animal Technology Co., Ltd	N/A

**Oligonucleotides**		

Primer: GAPDH (F): 5′- AGGTCGGTGT GAACGGATTTG -3’ (R): 5’ - TGTAGAC CATGTAGTTGAGGTCA -3′	This paper	N/A
Primer: M (F): 5′- GACCRATCCTGTCA CCTCTGAC -3’ (R): 5’ - GGGCATTYTG GACAAAKCGTCTACG -3′	This paper	N/A
Primer: CCL19 for q-PCR (F): 5′- CTGCT GGTTCTCTGGACTTCC -3’ (R): 5’ - AGG GATGGGTTTCTGGGTCA -3′	This paper	N/A
Primer: CCL19 for RT-PCR (F): 5′- ATGG CCCTGCTACTGGCCCTCA -3’ (R): 5’ - ACT GCTGCGGCGCTTCATCTT -3′	This paper	N/A
Primer: pHW2000 (F): 5′- CTAGCAGTT AACCGGAGTACTGGTCG -3’ (R): 5’ - GTTTTACGGCTGAGCCTCGCTTT -3′	This paper	N/A
Primer: Granzyme B (F): 5′- CCACTCT CGACCCTACATGG -3’ (R): 5’ - GGCC CCCCAAAGTGACATTTATT -3′	This paper	N/A
Primer: Perforin (F): 5′- GAGCTTCGTA GGGCCATGAC -3’ (R): 5’ - TCCATTA AGGACTGTTGCATCTG -3′	This paper	N/A
Primer: IFN-γ (F): 5′- ATGAACGCTA CACACTGCATC -3’ (R): 5’ - CCATC CTTTTGCCAGTTCCTC -3′	This paper	N/A
Primer: IL-2 (F): 5′- TGAGCAGGATGG AGAATTACAGG -3’ (R): 5’ - GTCCAA GTTCATCTTCTAGGCAC -3′	This paper	N/A

**Software and algorithms**		

GraphPad Prism9.3.1	GraphPad Prism Software	https://www.graphpad.com/
Adobe Illustrator 2022	Adobe	N/A
ImageJ	ImageJ Software	https://imagej.nih.gov/ij/

**Other**		

RPMI-1640 Medium	Gibco	C22400500BT
Fetal Bovine Serum	Newzerum	FBS-PA500
RPMI-DMEM Medium	Gibco	C11995500BT
DEPC-Treated Water	Biosharp	BL510B
Transwell Chamber	Corning	3422
Matrigel Matrix	Shanghai Novogene	0827215
10×Perm buffer	eBiocience	00-8333-56
Bouin’s Fixative Solution	Feijing Biology	PH0976

### Experimental model and study participant details

#### Ethics statement

All animal experiments were performed in strict accordance with the recommendations in Guidelines for Proper Conduct of Animal Experiments of Science Council of China. The protocol was approved by the Committee on the Ethics of Animal Experiments of Kunming University of Science and Technology (permit number: KUST-2021-05).

#### Mice

Eight-week-old female BALB/c mice were purchased from Vital River Laboratory Animal Technology Co., Ltd (Beijing, China). Animal welfare and experimental procedures were conducted strictly accordance with the Declaration of Helsinki and the recommendations in the Guide for the Care and Use of Laboratory Animals of the National Institutes of Health. All surgeries were performed under anesthesia, and great care was taken to minimize suffering.

#### Cell lines

Canine kidney cell line MDCK, monkey kidney cell line Vero, and human embryonic kidney cell line 293T were kindly provided by Ph.D. Liao Guoyang of Institute of Medical Biology, Chinese Academy of Medical Sciences. The mouse colon carcinoma cell line CT26, as well as the human colon carcinoma cell lines HT-29, HCT116, SW620, LoVo, and the normal human colonic mucosal epithelial cells line CCD841, were purchased from the American Type Culture Collection (ATCC, USA).

These MDCK, 293T, CCD841 cells were cultured with Dulbecco’s modified Eagle’s medium (DMEM) and other cells were cultured with RPMI 1640 medium. All medium were supplemented with 10% fetal bovine serum (FBS), and all cells were cultured at 37°C and 5% CO_2_ conditions.

### Method details

#### Construction of rPR8-CCL19

The recombinant CCL19 gene sequence, containing T2A, signal peptide, CCL19 and the signal packaging sequence at the 3′ terminal of PB1 of PR8, was obtained by gene synthesis, and was inserted into 3′ terminal of PB1 on the recombinant bidirectional plasmid pHW2000-PB1 which has been constructed previously in our laboratory. Then the pHW2000-PB1-CCL19 and other 7 recombinant bidirectional expression plasmids were co-transfected into 293T cells at 90% confluency following the principle of equal mixing. This new oncolytic influenza virus was named rPR8-CCL19.

#### RT-PCR indentification method

To detect the genetic passage stability of *r*PR8-CCL19, it used the EndoFree Midi Plasmid Kit (Tiangen, DP118). Conventional PCR was performed using PrimeSTAR Max DNA Polymerase (Takara, R045A) according to the manufacturer’s recommendations.

Their RNA was extracted using the TIANamp Virus RNA kit (Tiangen, DP315-R), cDNA was synthesized using Hifair AdvanceFast 1^st^ Strand cDNA Synthesis Kit (YEASEN, 11149ES60) and RT-PCR was performed using PrimeSTAR Max DNA Polymerase (Takara, R045A) according to the manufacturer’s recommendations.

For tumor tissue mRNA assay, total mRNA was extracted using the RNAprep Pure Tissue Kit (Tiangen, DP431), DNA was synthesized using Hifair Ⅱ 1^st^ Strand cDNA Synthesis SuperMix for qPCR (gDNA digester plus; YEASEN 11123ES60) and q-PCR was performed using Hieff qPCR SYBR Green Master Mix (YEASEN, 11201ES03) according to the manufacturer’s recommendations. All used primers were listed in Table 1.

**Table 1:** The used primers in this study

Primers	Sequences (5′→3′)
pHW2000 universal primer forward	CTAGCAGTTAACCGGAGTACTGGTCG
pHW2000 universal primer reverse	GTTTTACGGCTGAGCCTCGCTTT
CCL19 forward (for real-time PCR)	ATGGCCCTGCTACTGGCCCTCA
CCL19 reverse (for real-time PCR)	ACTGCTGCGGCGCTTCATCTT
GAPDH forward	AGGTCGGTGTGAACGGATTTG
GAPDH reverse	TGTAGACCATGTAGTTGAGGTCA
M forward	GACCRATCCTGTCACCTCTGAC
M reverse	GGGCATTYTGGACAAAKCGTCTACG
Granzyme B forward	CCACTCTCGACCCTACATGG
Granzyme B reverse	GGCCCCCAAAGTGACATTTATT
IFN-γ B forward	ATGAACGCTACACACTGCATC
IFN-γ B reverse	ATGAACGCTACACACTGCATC
Perforin forward	GAGCTTCGTAGGGCCATGAC
Perforin reverse	TCCATTAAGGACTGTTGCATCTG
IL-2 forward	TGAGCAGGATGGAGAATTACAGG
IL-2 reverse	GTCCAAGTTCATCTTCTAGGCAC
CCL19 forward (for qPCR)	CTGCTGGTTCTCTGGACTTCC
CCL19 reverse (for qPCR)	AGGGATGGGTTTCTGGGTCA

#### Virus titter calculation

To assess the replication ability and infection titter of *r*PR8-CCL19, hemagglutination assay and median tissue culture infective dose (TCID_50_) was performed according to our previous research.^43^

#### Cell viability testing

The CellTiter-Glo Assay was performed to assess cell viability using the CellTiter-Glo Luminescent Cell Viability Assay Kit (Promega, G7571) according to the manufacturer’s guidelines.

xCELLigence Real-Time Cell Analysis (RTCA) was carried out to monitor real-time dynamic cell proliferation using the xCELLigence RTCA DP instrument (Agilent, CA, USA) according to the manufacturer’s guidelines. Herein, RTCA was a label-free, dynamic method that continuously monitored cellular responses (e.g., proliferation, adhesion, or cytotoxicity) via impedance measurements. Signal drops reflected cell death or inhibited adhesion.

#### ELISA detection

The human CCL19 ELISA kit (Catalog No. HUEB0059), purchased from Beijing Sizhengbai Biotechnology Co., Ltd., was used to evaluate the expression levels of CCL19 in the culture supernatant of the recombinant oncolytic influenza virus rPR8-CCL19 across passages 1 to 5.

The assay was performed in strict accordance with the manufacturer’s instructions. Briefly, the culture supernatant of rPR8-CCL19 was collected and centrifuged at 1,000g for 10 min at 4°C to remove particulate matter. Serial dilutions of the provided standards were prepared to generate concentrations of 1000, 500, 250, 125, 62.5, 31.25, and 15.625 pg/mL. A standard curve was constructed using GraphPad Prism 9.3.1, and the concentration of CCL19 in the samples was determined based on the curve.

The kit utilizes a sandwich ELISA format. A monoclonal antibody specific to human CCL19 was pre-coated onto the microplate. Upon addition of standards or samples, any present CCL19 bound to the immobilized antibody. A biotinylated detection antibody against human CCL19 was then added, forming an antibody-antigen-antibody sandwich complex. Next, horseradish peroxidase (HRP)-conjugated streptavidin was introduced, which bound to the biotinylated antibody. After the addition of the tetramethylbenzidine (TMB) substrate, HRP catalyzed the conversion of TMB to a blue product, which turned yellow after acidification with the stop solution. The absorbance was measured at 450 nm using a microplate reader. The concentration of CCL19 in the samples was proportional to the absorbance and was interpolated from the standard curve.

#### Electron microscopy

To identify the morphological structure of *r*PR8-CCL19, the viral morphologies and sizes were observed using transmission electron microscopy after the virus was fixed and negatively stained with 2% phosphotungstic acid.^44^^,^^45^^,^^46^^,^^47^

#### Flow cytometry

Cell apoptosis was detected to evaluate the viral killing effect on tumor cells *in vitro*. The cells infected with virus (MOI = 0.01) for 24 h, 48 h 72 h were collected, respectively, and single-cell suspensions were prepared for apoptosis detecting using the Annexin V-FITC/PI Apoptosis Detection Kit (YEASEN, 40302ES20) according the manufacturer’s guidelines.

For evaluating the proliferation of immune cells, splenocytes were collected and prepared as single-cell suspension. The samples were then stained with different antibodies as follows: fixable viability stain 700 for dead cell preclusion, *tube 1:*CD3-PE, CD4-PerCP, CD8-APC, CD49b-FITC; *tube 2*: CD45-FITC, CD11b-PE, F4/80-APC, I-A/I-E-BV421, Ly6G-APC-cy7, Ly6C-Precp-CY5.5, CD11c-PE; *tube 3*: CD4-FITC, CD25-PE, Foxp3-APC. Antibodies were used to mark different immune cells for analysis needing.

#### Mitochondrial membrane potential, ΔΨm

The experiment cells Δψm was assessed using commercial kits (Beyotime Biotechnology, C2005). Briefly, after HT29 infected with virus (MOI = 0.01) for 24 h, these cells were washed with culture medium and incubated with fluorescent probe JC-1 (20 min) at 37°C in the dark. The fluorescent-labeled cells were washed again and detected via flow cytometry.

#### Western blotting

HT29 and CT26 cells infected with virus (MOI = 0.01) for 48 h were subjected to three freeze-thaw cycles at −80°C followed by centrifugation at 12,000 rpm for 10 min. Supernatants were analyzed using western blotting. The primary antibodies were used as follows: rabbit anti cleaved caspase3(CST, 9661T), rabbit anti pro-caspase3(Abclonal, a19654), rabbit anti-caspase9/pro-caspase9(CST, 9502T), rabbit anti-PARP (CST,9532), mouse anti-α-Tubulin (Abclonal,AC012); the secondary antibodies were used as follows: goat anti-Mouse IgG-HRP antibody (Servicebio, GB23301), goat anti-Rabbit IgG-HRP antibody (Servicebio, GB23303).

#### DCs activation *in vitro*

After treating CT26 cells with virus (MOI = 0.01) or *r*CCL19(1,000 ng/mL) for 48 h, the supernatant was collected and used to stimulate *i*DCs for 24 h. Subsequently, the markers, including CD80, CD86 and MHC-II which represent the maturation of DCs (*m*DCs), was analyzed using flow cytometry.

#### *i*DCs induction method

Immature dendritic cells (*i*DCs)were generated by culturing bone marrow cells collected from the femurs of BALB/c mice in DC medium (RPMI 1640 medium supplemented with 10% FBS, 20 ng/mL GM-CSF and 10 ng/mL IL-4). On the 7^th^ day, non-adherent and loosely adherent *i*DCs were collected, and their phenotype was determined by evaluating the expression of CD11c^+^ (60–80% CD11c^+^ cells were routinely obtained).

#### Transwell migration assay

To assess the chemotactic activity of CCL19 expressed by *r*PR8-CCL19, transwell assay was conducted. After CT26 cells were treated with virus (MOI = 0.01) or *r*CCL19(1,000 ng/mL) for 72 h, supernatants were added into the lower chambers of transwell plates, 800 μL/well. In the upper chambers, immune cells such as *i*DCs and immortalized macrophages were seeded, 4×10^4^ cells/well. The cells were observed through the upright microscope after 24 h.

#### Treatment and sampling in model animals

5×10^5^ CT26 cells were suspended in 75 μL PBS and subcutaneously implanted into right posterior flanks of BALB/c mice. Tumor volumes were measured using a digital caliper every other day and calculated using the following formula: Volume = length × width. When the tumor volume reached 70–100 cubic millimeter, the mice were randomly divided into four groups (*n* = 8): the PBS group treated by 10 μL PBS per mouse; the *wt*PR8 group treated by 1 × 10^5^ PFU *wt*PR8 per mouse; the *r*PR8-CCL19 group treated by 1 × 10^5^ PFU *r*PR8-CCL19 per mouse; the *r*CCL19 group treated by 0.2 μg recombinant protein CCL19 per mouse. Each mouse received a 10 μL injection. The mice were treated every other day for a total of 5 times and were sacrificed when tumor volumes exceeded 2,000 cubic millimeter. And it taken intratumor administration but not intranasal for all experimental mouse.

To investigate the inhibitory effect of *r*PR8-CCL19 in metastasis of CRC cells, these CT26 cells in the logarithmic growth phase were infected with virus (MOI = 0.01) or treated with *r*CCL19(1,000 ng/mL) before mouse model of tumor cells injection. After 2 h intervention, it prepared into single-cell suspension, subsequently, 100 μL of 1×10^5^ cells were injected through the tail vein per mouse. Two weeks later, lung tissues were collected and stained by Bouin’s fixative solution.

To evaluate the effect of systemic anti-tumor immune activation induced by *r*PR8-CCL19, bilateral CT26 models were prepared. A total of 5× 10^5^ CT26 cells were subcutaneously inoculated into the right posterior flank, and 2.5×10^5^ CT26 cells was inoculated into the left posterior flank simultaneously. Once the tumors on the right flank reached 70–100 cubic millimeter, unilateral intratumoral treatment was commenced as described above.

In re-challenge study, BALB/c mice with complete tumor regression after *r*PR8-CCL19 treatment and age-matched treatment-naïve female BALB/c mice were subcutaneously inoculated with 5×10^5^ CT26 cells in right posterior flanks to detect the activation of anti-tumor immune memory induced by *r*PR8-CCL19.

#### Hemagglutination inhibiton assay

All exprimental mouses’ blood were collected and then centrifuged at 12,000 rpm (4°C) for 10 min to obtain serum by collecting the supernatant. The serum was then mixed with four times the volume of receptor destroying enzyme (RDE) solution and incubated at 37°C for 18 h. Subsequently, the mixture was inactivated in at 56°C water bath for 45 min. Meanwhile, the influenza virus was diluted with physiological saline to achieve four hemagglutinating units. In a 96-well U-bottom microplate, 2-fold dilutions of serum (ranging from 1:10 to 1:5120) were prepared, with 25 μL of each diluted serum aliquot dispensed per well. Each well was then supplemented with 25 μL of 4 unite HA virus solution. Finally, 50 μL of 1% chicken red blood cell suspension was added to each well and thoroughly mixed. The plate was maintained at room temperature for 30 min before result interpretation.^46^^,^^47^^,^^48^

#### HE staining for animal tissues

To evaluate the safety of the *r*PR8-CCL19, histopathological changes with the liver, kidney, spleen, lung, and colon of mice were diagnosed through H&E staining according to standard protocols.^49^^,^^50^

#### IHC and *m*IF staining

To detect immune cells infiltration into TME, multi-color immunofluorescence (*m*IF) staining was performed. Tumor slides were stained successively with primary antibodies, fluorescein-labeled secondary antibodies, and then counterstained with DAPI according to standard process. The panoramic section scanner (3dhistech pannoramic desk/midi/250/1000) was used to collect images, and the indica labs-highplex FL v3.1.0 module of Halo v3.0.311.314 software was used to analyze the number of positive cells, colocalized cells, and total cells in each section.

For detecting the activity of TILs, IHC was used to analysis the expression of effector molecules, including Granzyme B, perforin and IFN-γ according to standard protocols. The mean optical density value was obtained by ImageJ software to quantify the expression level of effector molecules. All antibodies used above were listed in Table 2.

**Table 2:** The used antibodies for testing in this study

Used antibodies	Details of antibodies	Cat.No.
Primary antibodies	rabbit anti CD8	Servicebio, GB15068
	rabbit anti CD4	Servicebio, GB15064
	mouse anti CD3	Servicebio, GB12014
	mouse anti-Granzyme B	Servicebio, GB12092
	rabbit anti-perforin	ABclonal, A0093
	mouse anti-IFN-γ	Thermo Fisher Scientific, MM700
Secondary antibodies	CY5 labeled goat anti-rabbit IgG	Servicebio, GB25303
	CY3 labeled goat anti-rabbit IgG	Servicebio, GB21303
	Alexa Fluor 488 labeled goat anti-mouse IgG	Servicebio, GB25301
	polyclonal goat anti-mouse IgG labeled by HRP	Servicebio, GB23301
	polyclonal goat anti-rabbit IgG labeled by HRP	Servicebio, GB23303

##### Equipment

The key instruments used in this study are as follows: Flow cytometer: Agilent NovoCyte Advanteon (1–3 Lasers) Flow Cytometer (Agilent Technologies, Santa Clara, CA, USA); Real-time cell analysis system: xCELLigence RTCA DP Instrument (Agilent Technologies, Santa Clara, CA, USA); Transmission electron microscope (TEM): Hitachi HT7800 Transmission Electron Microscope (Hitachi High-Technologies Corporation, Tokyo, Japan); Microplate reader: Molecular Devices SpectraMax iD5e Multimode Microplate Reader (Molecular Devices, San Jose, CA, USA); Gel imaging system: Thermo Fisher Scientific iBright CL1500 Imaging System (Catalog Number: 44114; Thermo Fisher Scientific, Waltham, MA, USA); Conventional PCR system: Bio-Rad T100 Thermal Cycler (Catalog Number: 1861096; Bio-Rad Laboratories, Hercules, CA, USA); Quantitative real-time PCR (qPCR) system: Bio-Rad CFX Connect Real-Time PCR Detection System (Catalog Number: 1855200; Bio-Rad Laboratories, Hercules, CA, USA); Nucleic acid electrophoresis system: Servicebio SVL-2 Integrated Horizontal Electrophoresis System (Servicebio Technology Co., Ltd., Wuhan, China); Vertical electrophoresis system: Servicebio SVE-4 Integrated Vertical Electrophoresis System (Servicebio Technology Co., Ltd., Wuhan, China); Centrifuge: Servicebio SLX-1024F High-Speed Refrigerated Centrifuge (Servicebio Technology Co., Ltd., Wuhan, China); Inverted microscope: Olympus IXplore IX85 Automated Inverted Microscope System (Olympus Corporation, Tokyo, Japan); Digital pathology scanner: 3D Histech Pannoramic SCAN Fully Automated Digital Pathology System (3D Histech Kft., Budapest, Hungary).

##### Chemicals reagents

The key chemical reagents used in this study are as follows: RPMI-1640 Medium, Gibco (Thermo Fisher Scientific, Waltham, MA, USA), Catalog Number: C22400500BT; RPMI-DMEM Medium, Gibco (Thermo Fisher Scientific, Waltham, MA, USA), Catalog Number: C11995500BT; Fetal Bovine Serum (FBS), Newzerum (Newzerum Biotechnology Co., Ltd., China), Catalog Number: FBS-PA500; Polyfect Transfection Reagent, Qiagen (Qiagen N.V., Hilden, Germany), Catalog Number: 301105; 2×Taq Mix, Vazyme (Vazyme Biotech Co., Ltd., Nanjing, China), Catalog Number: P213-01/02/03; Matrigel, Shanghai Nova (Shanghai Nova Biotechnology Co., Ltd., Shanghai, China), Catalog Number: 0827215; DAPI Staining Reagent, Servicebio (Servicebio Technology Co., Ltd., Wuhan, China), Catalog Number: G1012; Eosin Staining Solution, Servicebio (Servicebio Technology Co., Ltd., Wuhan, China), Catalog Number: G1001-500 ML; Hematoxylin Staining Solution, Servicebio (Servicebio Technology Co., Ltd., Wuhan, China), Catalog Number: G1004-500 ML; DAB Substrate Kit, Abcam (Abcam plc, Cambridge, UK), Catalog Number: ab64238; EDTA, Biosharp (Biosharp Life Sciences, Hefei, China), Catalog Number: BS107-100g; Tris-base, Solarbio (Solarbio Science and Technology Co., Ltd., Beijing, China), Catalog Number: 77-86-1; 4×Fix/Perm Buffer Kit, eBioscience (Thermo Fisher Scientific, Waltham, MA, USA), Catalog Number: 88-8824-00; 10×Perm Buffer, eBioscience (Thermo Fisher Scientific, Waltham, MA, USA), Catalog Number: 00-8333-56; Bouin’s Fixative Solution, Dalian Meilun Biotechnology (Dalian Meilun Biotechnology Co., Ltd., Dalian, China), Catalog Number: PWL226; TPCK-Treated Trypsin, Merck (Merck KGaA, Darmstadt, Germany), Catalog Number: 4370285; RDE Solution, Kitasato Institute (Kitasato Institute for Life Sciences, Tokyo, Japan), Catalog Number: 240122. Other routine chemical reagents used in the study were purchased from Xilong Scientific Co., Ltd. (Xiamen, Fujian, China).

### Quantification and statistical analysis

All experiments were repeated three times independently at least. All statistical analyses were performed using GraphPad Prism9.3.1. All sample sizes and statistical methods were indicated in the corresponding figure legends. Data were analyzed using unpaired two-sided Student’s test, one-way ANOVA and two-way ANOVA for multiple comparisons. Survival was analyzed by the Kaplan–Meier method. The bars show the mean ± SD values. Significant differences were indicated as ∗*p* < 0.05, ∗∗*p* < 0.01, ∗∗∗*p* < 0.001, ∗∗∗∗*p* < 0.0001.
