# Supplementary material for: CCL19-armed recombinant influenza virus inhibited colorectal cancer growth by remodeling tumor microenvironment
Source: iScience. 2025 Nov 19;28(12):114127. doi: 10.1016/j.isci.2025.114127 (PMC12723135; doi:10.1016/j.isci.2025.114127)
Supplement: Document S1. Figures S1–S4 [file mmc1.pdf]

iScience, Volume *28*

Supplemental information

CCL19-armed recombinant influenza virus inhibited colorectal cancer growth by remodeling tumor microenvironment

Xia Ou, Yunxin Xia, Zhongyue Fang, Kai Yang, Guangtao Yang, Junying Wang, Yiyong Duan, Xiahui Yang, Bing Yang, Ze Liu, and Jihong Zhang

Supplemental figures and legends

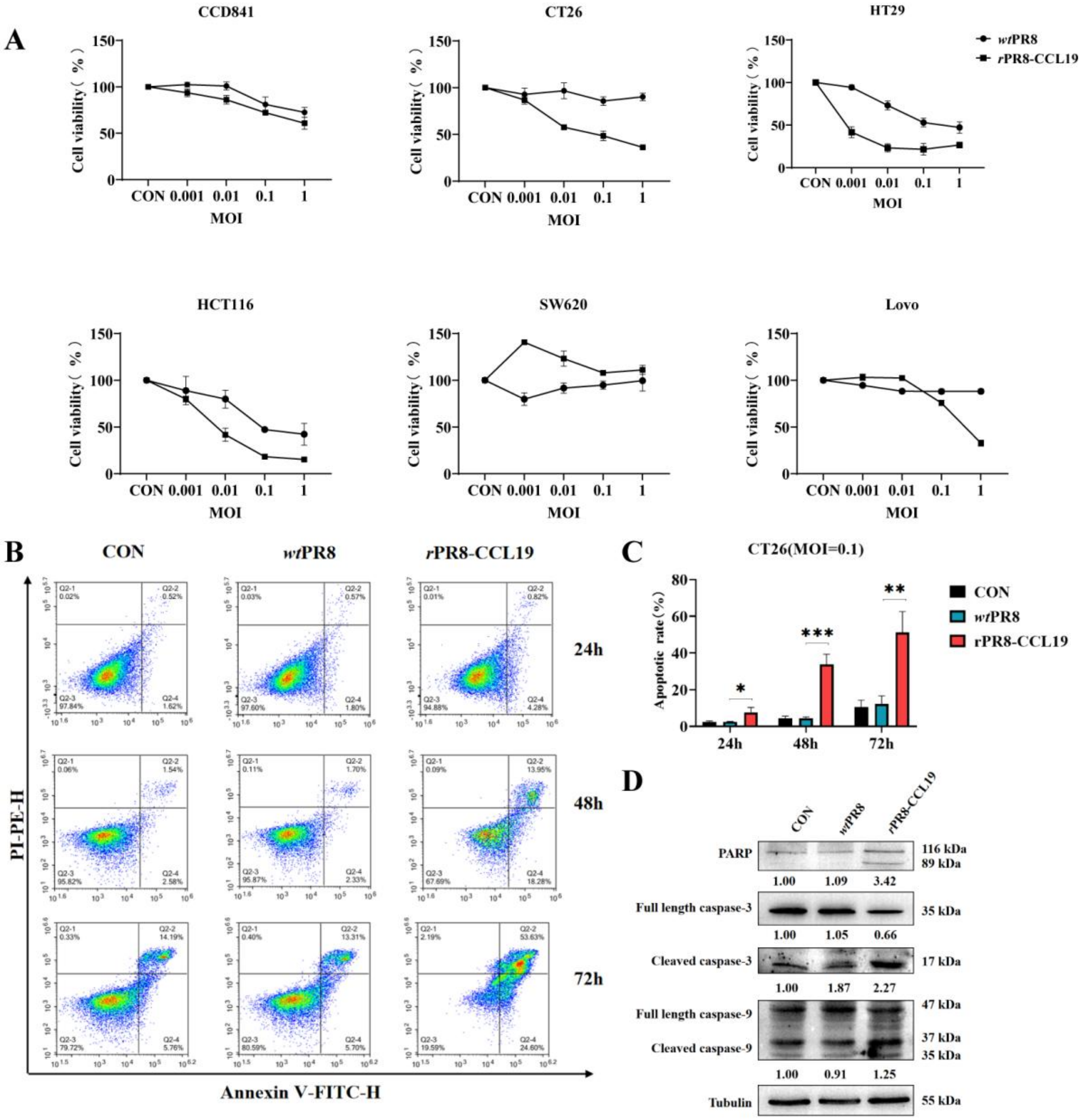

Supplementary Figure 1. The replication and tumor cell-killing activity of rPR8-CCL19 in vitro.

(A) Cell viabilities were measured by Cell Titer-Glo Assay after various CRC cells infected with rPR8-CCL19 and wtPR8;

(B) The flow cytometry analysis was used to detection of CT26 cell apoptosis after rPR8-CCL19 infection;

(C) Quantification of CT26 cell apoptotic ratio. Mean: bars ± SEM. n=3 in each group. (*$p < 0.05$, **$p < 0.01$, ***$p < 0.001$);

(D) The expressions of PARP, caspase-3, caspase-9, and their corresponding cleavers in CT26 cells infected rPR8-CCL19 and wtPR8 were detected by western blotting.

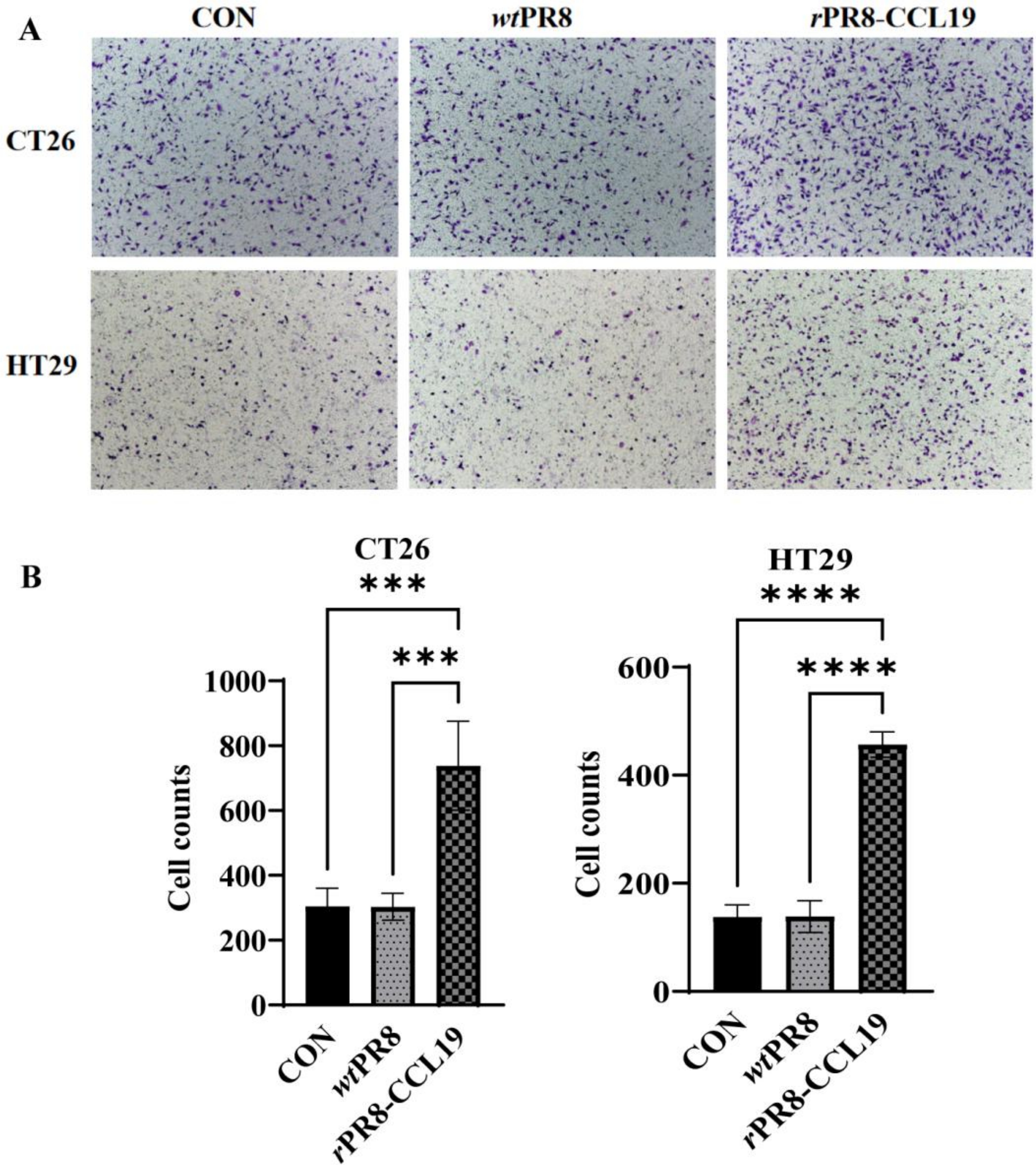

Supplementary Figure 2. The rPR8-CCL19 expressed CCL19, chemotacticed immune cells and activated them in vitro.

(A) Chemotactic effect of CCL19 secreted by rPR8-CCL19-infected CT26/HT29 cells on immortalized macrophages RAW264.7 was detected by transwell assay;

(B) Quantification of the numbers of RAW264.7 migrating to the lower chamber, rPR8-CCL19 was at singifidcant high degree.

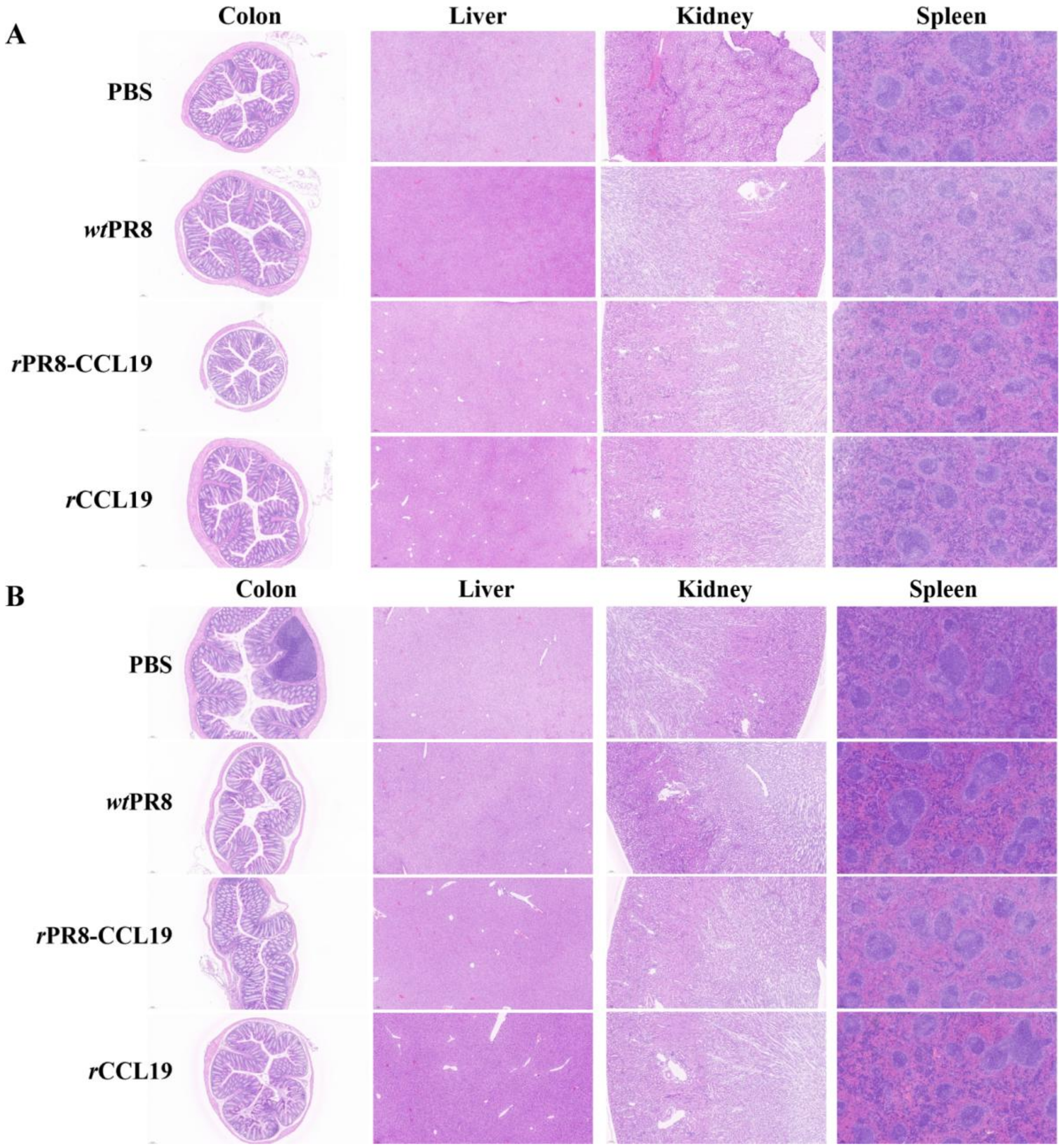

Supplementary Figure 3. The histological sections of treatment group mouse with different important tissues to evaluate rPR8-CCL19 safety, including liver, kidney and spleen. (A) The histological sections of treatment group mouse different tissues at 11th day, it was stained by H&E. rPR8-CCL19 couldn’t cause pathological injury. Scale bars, 100 μm; (B)The histological sections of treatment group mouse different tissues at 21st day, it was stained by H&E. rPR8-CCL19 couldn’t cause pathological injury. Scale bars, 100 μ

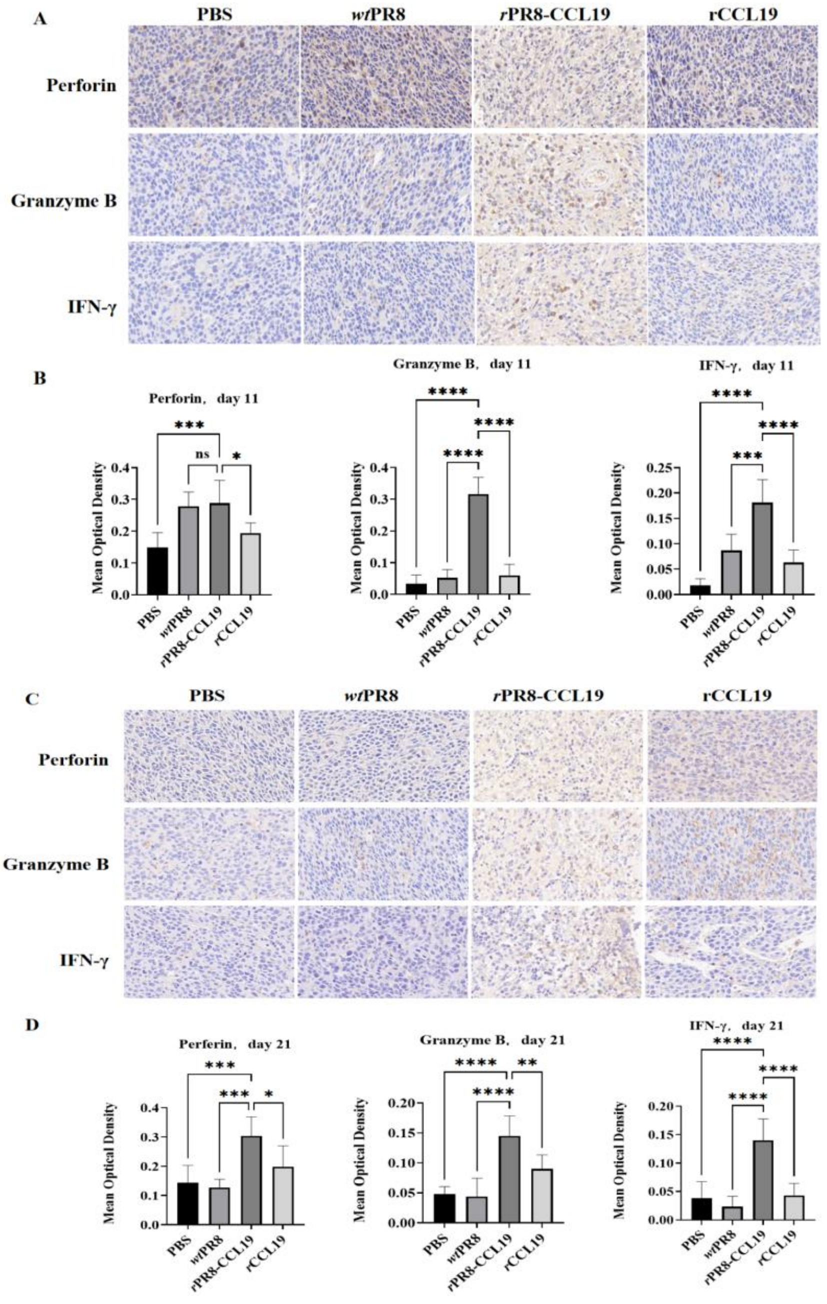

Supplementary Figure 4. The tumor microenvironment was remodeled by rPR8-CCL19 virus after oncolytic treatment in vivo, compared with other groups.

(A) IHC was performed to detecting the expressions of pro-inflammatory factors and inflammatory factors, including Granzyme B, perforin, and IFN-γ in TME, respectively, at 11th day after OV therapy; (B) The recruitment of inflammatory factors was quantified using bar graph analysis. The rPR8-CCL19 group exhibited a higher activation level, showing statistically significant differences compared to other groups, as analyzed on day 11 post-treatment;

(C) IHC was performed to detecting the expressions of pro-inflammatory factors and inflammatory factors, including Granzyme B, perforin, and IFN-γ in TME, respectively, at 21st day after OV therapy; (D) The recruitment of inflammatory factors was quantified using bar graph analysis. The rPR8-CCL19 group exhibited a higher activation level, showing statistically significant differences compared to other groups, as analyzed on day 21 post-treatment.
